# Dimethyl Fumarate Therapy Significantly Improves the Responsiveness of T Cells in Multiple Sclerosis Patients for Immunoregulation by Regulatory T Cells

**DOI:** 10.3390/ijms18020271

**Published:** 2017-01-28

**Authors:** Janine Schlöder, Carsten Berges, Felix Luessi, Helmut Jonuleit

**Affiliations:** 1 Department of Dermatology, University Medical Center of the Johannes Gutenberg-University, Langenbeckstr. 1, 55131 Mainz, Germany; jschloed@uni-mainz.de (J.S.); caberges@uni-mainz.de (C.B.); 2 Department of Neurology, University Medical Center of the Johannes Gutenberg-University, Langenbeckstr. 1, 55131 Mainz, Germany; luessi@uni-mainz.de

**Keywords:** multiple sclerosis, therapy, immune regulation, T effector cells, Treg resistance, dimethyl fumarate, humanized mice

## Abstract

Multiple sclerosis (MS) is a chronic autoimmune disease caused by an insufficient suppression of autoreactive T lymphocytes. One reason for the lack of immunological control is the reduced responsiveness of T effector cells (Teff) for the suppressive properties of regulatory T cells (Treg), a process termed Treg resistance. Here we investigated whether the disease-modifying therapy of relapsing-remitting MS (RRMS) with dimethyl fumarate (DMF) influences the sensitivity of T cells in the peripheral blood of patients towards Treg-mediated suppression. We demonstrated that DMF restores responsiveness of Teff to the suppressive function of Treg in vitro, presumably by down-regulation of interleukin-6R (IL-6R) expression on T cells. Transfer of human immune cells into immunodeficient mice resulted in a lethal graft-versus-host reaction triggered by human CD4^+^ Teff. This systemic inflammation can be prevented by activated Treg after transfer of immune cells from DMF-treated MS patients, but not after injection of Treg-resistant Teff from therapy-naïve MS patients. Furthermore, after DMF therapy, proliferation and expansion of T cells and the immigration into the spleen of the animals is reduced and modulated by activated Treg. In summary, our data reveals that DMF therapy significantly improves the responsiveness of Teff in MS patients to immunoregulation.

## 1. Introduction

Multiple sclerosis (MS) is a neurodegenerative, T cell-mediated inflammatory autoimmune disease caused by autoaggressive immune reactions against myelinated axons in the central nervous system (CNS), leading to their destruction and thus producing significant physical disability. Recent studies have identified a dysfunction of patient-derived regulatory T cells (Treg) and a dysregulation of effector T cells (Teff) as important key mechanisms in the course of disease [1,2,3]. We and others have demonstrated that Teff dysregulation is characterized by their unresponsiveness to the suppressive control of Treg, a mechanism termed Treg resistance [3,4].

Common therapeutic options for MS can be divided into two major groups: treatments for acute attacks (e.g., corticosteroids) and disease-modifying therapies for immunomodulation and suppression. Among all immune-modifying options, interferon-β (IFN-β) is the first-line treatment for patients with a relapsing-remitting MS (RRMS) [5,6]. In addition to its modulatory and immunosuppressive function by increasing interleukin-10 (IL-10) serum levels in RRMS patients [7], the influence of IFN-β on Treg resistance has been extensively studied by our group in the last few years. We recently showed that Teff from IFN-β-treated patients exhibit a restored responsiveness for immune suppression by Treg associated with a diminished IL-6R expression compared to therapy-naïve MS patients [8]. Even though IFN-β reduces the frequency and severity of clinical relapses, one major disadvantage of this treatment is still the induction of neutralizing anti-interferon antibodies, which diminished the clinical effect significantly [9,10]. Dimethyl fumarate (DMF), first included in the treatment of psoriasis, has become a promising emerging drug also for the treatment of RRMS in the last years [11,12,13]. Up to now, it is known that DMF diminishes neuroinflammation by promoting the cytoprotection of CNS cells against oxidative stress, which appear to be mediated mainly through the activation of Nrf2 pathway [14,15]. Furthermore, DMF also plays a role in modulating immune responses by inducing type II dendritic cells, which produce IL-10 instead of IL-12 and IL-23, and, therefore, attenuating pathogenic T cell development [16,17]. DMF induces a shift towards anti-inflammatory immune responses by directly inhibiting pro-inflammatory pathways [18] or decreasing frequencies of circulating Th1 cells [19,20]. However, the influence of DMF on Treg sensitivity of Teff has, so far, not yet been addressed.

In this study, we focused on influences of DMF therapy on T cell immune regulation. We demonstrated that DMF significantly restores the sensitivity of Teff in peripheral blood of MS patients for the immunoregulatory function of Treg. Thus, we identified a novel therapeutic immunoregulatory mechanism of DMF therapy.

## 2. Results

### 2.1. DMF Restores Treg Resistance of MS T Effector Cells In Vitro

In autoimmune diseases like MS, Treg are incapable of controlling the activation of autoreactive T cells. This phenomenon has been identified in different autoimmune diseases reasoned by impaired Treg function and unresponsiveness of Teff to immunoregulatory control mechanisms [21,22]. In the case of MS, T cells from therapy-naïve patients are widely insensitive to Treg-mediated suppression when compared to Teff from healthy controls (HC) [3,8]. Furthermore, we demonstrated that IFN-β therapy restores T cell responsiveness towards the suppressive capacity of Treg. Although IFN-β is an important therapeutic approach in MS, several side effects limit its activity, and its failure to control the progressive forms of MS has been reported.

DMF, a newly approved drug for the treatment of RRMS [12,23,24], has shown promising immune-modulatory effects. To investigate potential influences of DMF on immune regulation of T effector cells, we analyzed peripheral T cells from therapy-naïve and DMF-treated patients. Ten patients with RRMS showed a relapse, the remaining patients were in remission. Twenty-five were treated for at least four months with DMF (Tecfidera). Other patients had not received previous treatment six months before time point of analysis and were clinically stable (Table 1).

**Figure 1:**
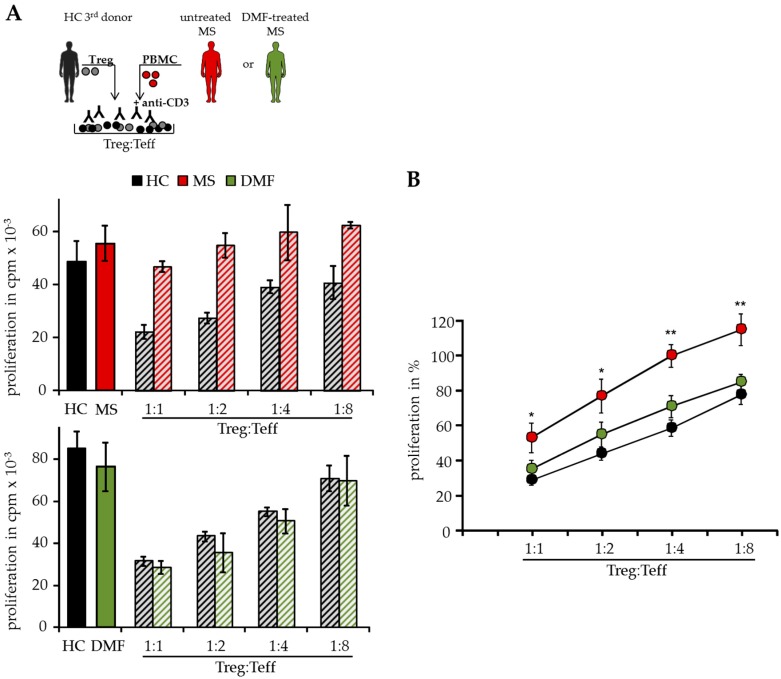
Dimethyl fumarate (DMF) restored T effector cell (Teff) responsiveness to wards the suppressive control of regulatory T cells (Treg). (**A**) Treg-depleted peripheral mononuclear cells (PBMC) from therapy-naïve multiple sclerosis patients (MS, red), DMF-treated patients (DMF, green) or healthy controls (HC, black) were cocultured with different ratios of Treg from independent third donors and polyclonal stimulated with 0.5 µg/mL anti-CD3 mAb. Stimulation in absence of Treg served as control. Proliferation was determined by ^3^H-Tdr incorporation on day three and displayed as mean counts per minute (cpm) ± standard deviation (SD) of triplicates. One representative experiment out of 15 is shown. (**B**) Relative proliferation in presence of different Treg ratios is shown as mean of eight independent experiments per group ± standard error of the mean (SEM), *p*-values of therapy-naïve MS patients compared to T cell proliferation of DMF-treated patients * *p* < 0.05, ** *p* < 0.01.

In T cell suppressor assays, we analyzed Teff function isolated from peripheral blood of different MS patients independent of patient intrinsic Treg. Therefore, polyclonal activated Treg-depleted PBMC were cocultured with isolated Treg from independent third healthy donors (HC, Figure 1A). As shown previously, activated Treg suppressed T cell proliferation donor-independently, but Treg-resistant Teff from therapy-naïve MS patients were insensitive to Treg-mediated suppression in these assays [8]. However, DMF therapy modulated the Treg sensitivity in treated patients and their Teff were efficiently suppressed by activated Treg comparable to HC, indicating that DMF overcomes the Treg resistance and restores the responsiveness of Teff for immunosuppression (Figure 1A,B).

### 2.2. Restored Treg Sensitivity of Teff from DMF-Treated MS Patients Correlates with a Downregulated IL-6R Expression

**Figure 2:**
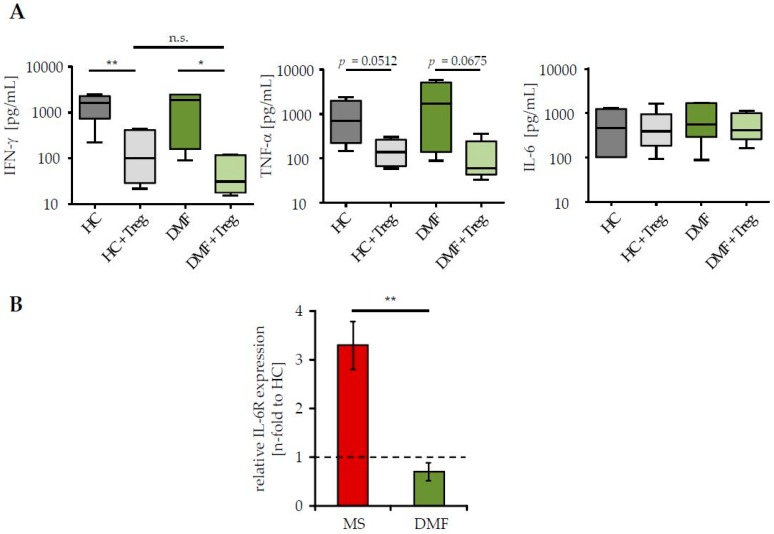
DMF therapy normalized interleukin-6R (IL-6R) expression on T cells of MS patients. (**A**) Supernatants of polyclonal activated PBMC (0.5 µg/mL anti-CD3 mAb, stimulation in presence/absence of Treg, ratio 1:1) were collected at day three and cytokine profiles were analyzed. Data represent pooled results of six donors per group (therapy-naïve red, after DMF therapy green) or HC (grey). Median and interquartile ranges are depicted, *p*-values relative to PBMC cocultured with Treg are shown * *p* < 0.05, ** *p* < 0.01, n.s. = not significant; (**B**) IL-6R expression on CD3^+^ T cells from therapy-naïve (red) or DMF-treated (green) patients was determined by flow cytometry. Data display fold change in IL-6R expression to HC (black dashed line), *p*-value relative to IL-6R expression of therapy-naive MS patients ** *p* < 0.01 is shown.

Treg resistance is directly associated to changes in IL-6 signaling, either by an enhanced IL-6R expression and/or by accelerated kinetics of IL-6 production; both reduce the efficiency of Treg-mediated suppression [3,4]. Since DMF therapy of MS patients restored their sensitivity to the suppressive control through Treg, we investigated whether this therapy affects IL-6 signaling of Teff. We measured IL-6 production in supernatants of Treg-depleted PBMC from patients and HC in presence or absence of activated Treg from third healthy controls. Analyses of Th1 cytokines IFN-γ and tumor necrosis factor-α (TNF-α), which are associated with MS progression and disease worsening, were included [25]. We observed a similar production of IFN-γ, TNF-α and IL-6 in stimulated Teff of patients after DMF therapy and HC (Figure 2A). In the presence of Treg, IFN-γ and TNF-α synthesis were considerably suppressed, reflecting their comparable Treg sensitivity. IL-6 production was not influenced by Treg-mediated T cell suppression. More interestingly, DMF therapy significantly reduced the expression of IL-6R on CD3^+^ T cells, suggesting that DMF therapy largely restored the disturbed T cell function by changes in IL-6 signaling (Figure 2B).

### 2.3. Improved Responsiveness of T Cells from DMF-Treated MS Patients to Treg-Mediated Suppression In Vivo

**Figure 3:**
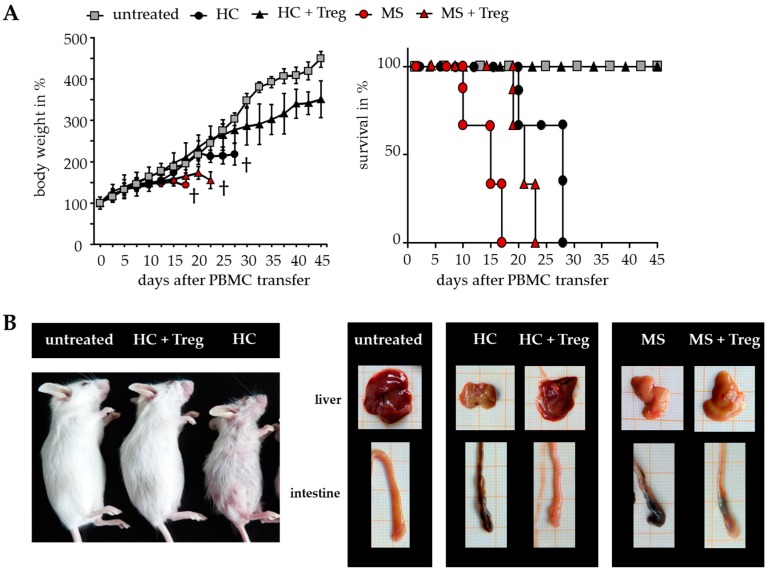
Teff from therapy-naïve MS patients induced a severe graft-versus-host disease (GvHD) without protection by activated Treg. (**A**) Treg-depleted PBMC from either MS patients or HC ± activated Treg from third healthy controls were transferred into newborn immunodeficient mice. Untreated mice served as controls. Each point represents the cumulative mean weight of one group ± SD of *n* = 5 mice in each group (**left**); survival of mice in Kaplan–Meier plot till day 45 is shown (**right**); (**B**) Shown are representative pictures of mice and abdominal organs 25 days after transfer; skin inflammation (**left**); clinical appearance of liver and intestine (**right**).

The results suggested that DMF is able to restore the disturbed T cell immune regulation in MS patients. However, only a few parameters of Treg-mediated suppression can be analyzed after polyclonal T cell stimulation in vitro. To overcome the limitations, additional investigations were performed in humanized mice in vivo. Transfer of human peripheral immune cells in immunodeficient mice induces a classical graft-versus-host disease (GvHD) associated with a loss of body weight and inflammation of the liver, skin and intestines. GvHD is triggered by CD4^+^ T helper cells, which differentiate in the animal to tissue-reactive and aggressive Teff. Cotransfer of a sufficient number of activated Treg can suppress the formation of GvHD, depending on the responsiveness of CD4^+^ T helper cells for Treg-mediated suppression. As shown before, Treg-resistant CD4^+^ T cells from therapy-naïve MS patients are insensitive to Treg-mediated immunosuppression. Transfer of these T cells leads to an accelerated GvHD formation, characterized by severe symptoms of dermatitis, hepatitis and colitis, which cannot be prevented by Treg (Figure 3) [8,26].

Since the immigration of human CD4^+^ T cells into the spleen of mice is the first and essential step of GvHD induction, we investigated the splenic T cell infiltration after transfer of PBMC from therapy-naïve or DMF-treated patients and HC with/without additional Treg.

**Figure 4:**
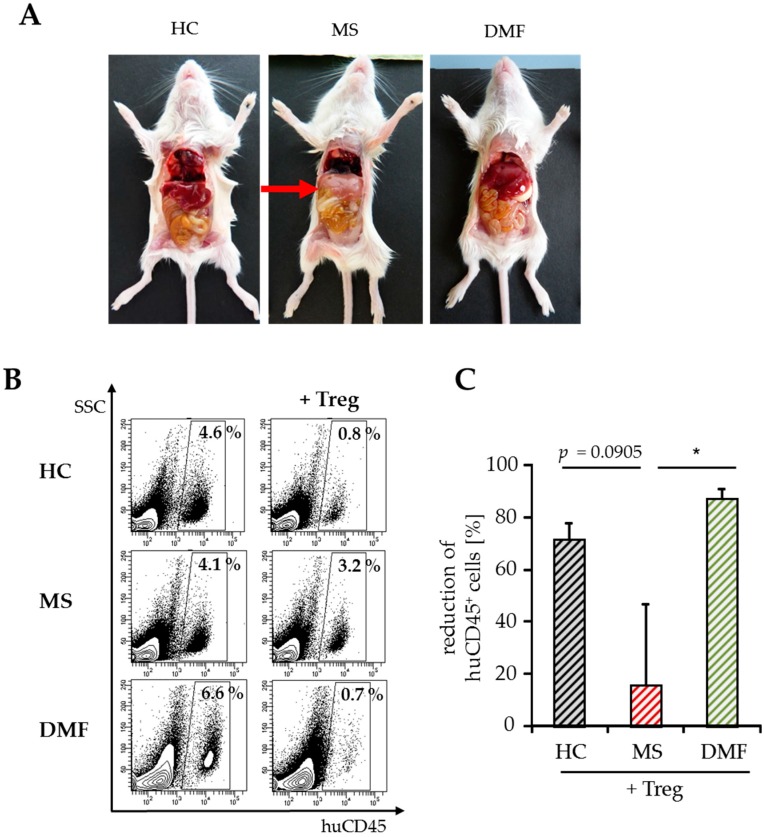
Analyses of splenic infiltrates and clinical symptoms after GvHD onset. (**A**) Abdominal organs (liver, intestine) of mice eight days after transfer of human PBMC from therapy-naïve (MS) or DMF-treated patients (DMF) and HC. Untreated mice served as a control; (**B**) Data show a representative analysis of immigrated human CD45^+^ immune cells, SSC = sideward scatter; (**C**) Reduction of human splenic immune cell immigration by Treg. Data are displayed as mean values ± SEM of *n* = 5 mice per group and *p*-values relative to MS * *p* < 0.05.

For detailed analysis of T cell activation in vivo, we stopped the experiments shortly after GvHD onset (8–10 days after transfer) and investigated the amount of immigrated splenic human immune cells by flow cytometry. At this early time point, hepatitis, a characteristic clinical GvHD symptom, was only observed in mice engrafted with PBMC from therapy-naïve MS patients (Figure 4A). The whole number of spleen cells did not differ significantly between the groups ((30–50) × 10^6^, Table 2) after transfer of Treg-depleted PBMC with similar ratios of immigrated human immune cells. Cotransfer of activated Treg markedly reduced human immune cell infiltration up to 80% in mice engrafted with PBMC from DMF-treated patients or HC, but not after transfer of PBMC from therapy-naïve MS patients (Table 2, Figure 4B,C).

### 2.4. Treg-Reduced Amount of Activated CD4^+^ T Cells from DMF-Treated MS Patients In Vivo

**Figure 5:**
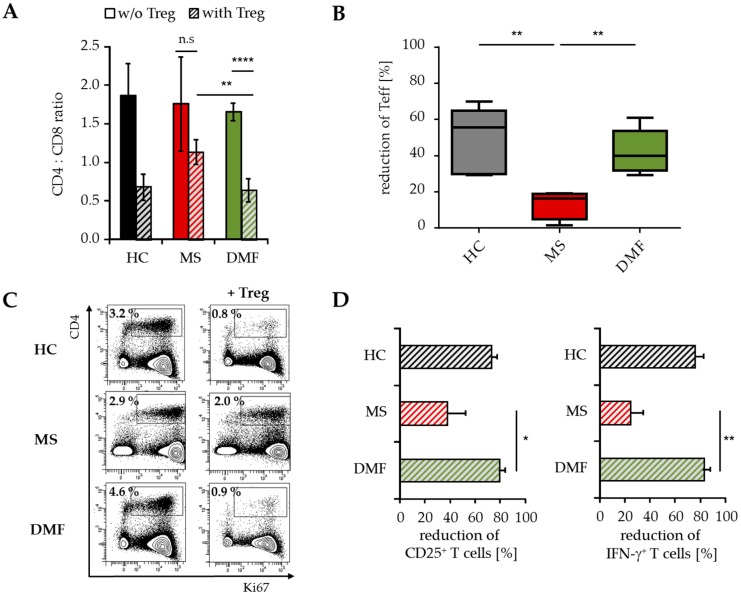
Analysis of T cell infiltrates modulated by Treg. Analysis of human T cells in the spleen of mice transferred with PBMC from therapy-naïve (MS, red) and DMF-treated MS patients (DMF, green) or HC (black) ± Treg, *n* = 5 mice in each group. (**A**) Data displayed as mean CD4:CD8 ratio ± SEM; ** *p* < 0.01, **** *p* < 0.0001, n.s. = not significant, w/o = without Treg; (**B**) Reduction of splenic human CD4^+^ T cells by Treg. Median and interquartile ranges are depicted, *p*-values relative to MS are shown ** *p* < 0.01; (**C**) Data representative for Ki67 expression on human CD4^+^ T cells; (**D**) Reduction of splenic CD25^+^ and IFN-γ^+^ CD4^+^ T cells by Treg, displayed as mean values ± SEM, *p*-values relative to MS * *p* < 0.05, ** *p* < 0.01, IFN = interferon.

Flow cytometric analyses demonstrated a reduced CD4:CD8 ratio in the spleen of mice transferred with PBMC from DMF-treated versus therapy-naïve MS patients (Figure 5A). This was attributed to a considerably reduced percentage of immigrated human CD4^+^ Teff up to 40%–60% (Figure 5B). Additional investigations regarding the proliferation and activation status of infiltrated T cells showed decreased ratios of CD25^+^, Ki67^+^ and IFN-γ-producing CD4^+^ Teff (Figure 5C,D), indicating that Treg suppressed the inflammatory T cell response in vivo mainly by reducing the number of activated CD4^+^ T cells.

## 3. Discussion

In the current study, we investigated the effect of DMF therapy on T cell responsiveness of RRMS patients to Treg-mediated suppression. In accordance with our previous publications, we observed that Teff from therapy-naïve MS patients are predominantly insensitive to the suppressive control of Treg. In the case of MS, this Treg resistance is strongly associated with impaired IL-6 signaling [3,4,26]. DMF is a newly approved drug for RRMS whose mechanism of action has not been fully resolved so far [23,24]. Here we show that DMF treatment influences Treg resistance, a key pathologic mechanism in MS patients, by normalizing elevated IL-6R expression and thus restoring susceptibility of T effector cells to Treg-mediated suppression.

In recent years, compelling evidence has been found suggesting that dysregulated T cell homeostasis plays an essential role in the pathogenesis of autoimmune diseases like rheumatoid arthritis, type 1 diabetes and MS [27,28,29]. This dysregulation is caused by T cells that become refractory to Treg-mediated suppression and other mechanisms of the immune tolerance network. Many resistance-inducing mechanisms have been described, indicating that extracellular factors and the cytokine milieu (e.g., IL-6, TNF-α) are responsible for impaired susceptibility of T cell subsets to Treg suppression [4,30,31]. Since many immune-modulatory drugs are used as first-line therapies in MS, it is crucial to investigate whether these drugs affect cytokine production and thus Treg resistance. For IFN-β treatment, a dominant immunosuppressive effect on T cell activation and IFN-γ production [32] has been reported. Furthermore, IFN-β therapy of RRMS patients significantly improves T cell responsiveness towards Treg-mediated suppression [8]. This effect strongly correlated with IL-6R down-regulation, thereby inhibiting the IL-6 signaling pathway. Similarly, DMF-mediated improvement of T cell responsiveness was also strongly associated with reduced IL-6R expression on T cells. This is in line with studies demonstrating that DMF inhibited proliferation and induces apoptosis in T cells [33,34].

A variety of autoimmune diseases are associated with high levels of IL-6 production [35,36]. Therefore, it is crucial to investigate whether immune-modulatory drugs such as IFN-β and DMF might affect IL-6 expression. Clinical studies revealed that IFN-β treatment significantly reduces IL-6 serum levels in RRMS patients [37,38]. Furthermore, in vitro studies showed neuroprotective effects of DMF by decreasing the production of inflammatory cytokines, such as IL-6, in microglia and astrocytes, indicating that DMF might inhibit the expression of neuroinflammatory mediators in the brain of MS patients [39]. Thus, DMF and IFN-β might exert their beneficial effect by targeting IL-6 signaling in Teff, highlighting the crucial role of the IL-6 signaling pathway in MS.

Increased levels of pro-inflammatory Th1 cytokines like TNF-α and IFN-γ were observed in MS patients during relapse [25,40,41,42]. We found no significant differences in IFN-γ and TNF-α synthesis in activated Teff of DMF-treated patients and HC, suggesting that DMF therapy normalizes Th1 cytokine production in patients. This is in agreement with observations that showed a reduction of Th1-type T cells in the course of DMF therapy [19,43]. Furthermore, polyclonal activated Treg reduced IFN-γ and TNF-α in Teff from DMF-treated MS patients comparable to HC, emphasizing again that DMF restores the responsiveness of Teff for Treg-mediated suppression.

Adoptive transfer of human PBMC into newborn immunodeficient mice leads to the induction of GvHD triggered by human CD4^+^ T effector cells. Disease is suppressed or prevented by efficient ratios of activated Treg [44,45]. This humanized mouse is an appropriate model to study the regulation and modulation of human T cell responses in vivo, especially from rare patient-derived immune cells. As previously shown, transferring PBMC from therapy-naïve MS patients into newborn mice resulted in an accelerated GvHD, which cannot be prevented or suppressed by Treg. Since the GvHD is an antigen-specific, systemic and chronic immune response, this mouse model is useful to investigate and modulate basic parameters of the immune regulatory network in vivo. To clarify the effects of DMF therapy on MS T cells, we analyzed human T cells ex vivo shortly after GvHD onset. In accordance with the in vitro data, Treg significantly suppressed proliferation and expansion of CD4^+^ T effector cells from DMF-treated MS patients and thus prevented lethal and clinical symptoms of GvHD in vivo.

Since DMF restores predominantly T cell sensitivity to Treg control, occasional cases of relapse in DMF-treated MS patients cannot be excluded. Further studies must show whether immune-modulatory drugs such as IFN-β and DMF might influence the disturbed function of Treg in MS patients. In addition, MS is a multifactorial autoimmune disease that is caused by a complex interaction between immune cells and tissue, thereby contributing to the pathological heterogeneity. In our opinion, a combined treatment approach addressing T cell responsiveness and Treg dysfunction might be beneficial.

## 4. Materials and Methods

### 4.1. Patients and Healthy Controls

Forty-nine patients with a relapsing–remitting course (RRMS, age 18 to 63 years) and three with a clinically isolated syndrome (CIS, age 41 to 48 years), fulfilling the revised McDonald criteria for multiple sclerosis [36], were included in this study. Ten patients with RRMS showed a relapse, the remaining patients were in remission. Twenty-five patients were treated for at least four months with DMF (Tecfidera). Other patients had not received previous treatment or immunosuppressive agents six months before time point of analysis and were clinically stable. PBMC from healthy donors (HC) served as controls. All PBMC were isolated within 12 h after blood collection by Ficoll density gradient centrifugation and were used directly in experiments. According to the principles expressed in the Helsinki Declaration and to the ethics committee-approved protocols (Landesärztekammer Rhineland Palatine No. 837.019.10 (7028), approved on 4 March 2010), patients provided written informed consent before participating in this study.

### 4.2. Transfer of Human Immune Cells

Rag2^−/−^γc^−/−^ mice [46] were obtained one to four days after birth from the central animal facility (Translational Animal Research Center, Johannes Gutenberg-University, Mainz, Germany). Experiments were performed in accordance with relevant laws and institutional guidelines. GvHD was induced as described before [47]. Briefly, 5 × 10^6^ CD25-depleted PBMC from HC, therapy-naïve or DMF-treated MS patients were injected intraperitoneally into newborn mice with/without 5 × 10^5^ activated Treg (5 µg gp120 per mouse) from independent healthy controls. Untreated mice served as controls. Body weight was measured every second day and animals with severe symptoms were killed [48]. Results are presented as percent mean body weight ± SEM based on initial weight. For T cell analysis, mice were sacrificed 8–10 days after transfer and spleen cells were collected for further analysis ex vivo.

### 4.3. Culture Medium and Antibodies

Peripheral human immune cells and immune cells re-isolated from spleens of humanized mice were cultured in X-VIVO-15 (Lonza, Verviers, Belgium). Flow cytometric analysis was performed using the following antibodies. Anti-human CD3 (SK7), anti-human CD3 (UCHT1), anti-human CD4 (RPA-T4), anti-human CD8 (SK1) all from BD Pharmingen (Heidelberg, Germany); anti-human CD45, anti-human CD25, anti-human IL-6 receptor (REA291) from Miltenyi Biotec (Bergisch Gladbach, Germany). Cell viability during flow cytometric analyses was determined using fixable viability dye eFluor 506 (eBioscience, San Diego, CA, USA).

### 4.4. Flow Cytometry

For surface staining of PBMC or isolated T cells, antibodies were incubated for 30 min at 4 °C and washed twice with phosphate buffered saline (PBS). Stained cells were measured on LSRII with FACS Diva Software (Version 6.1.1, BD Biosciences, Heidelberg, Germany). For intracellular staining of proliferation marker Ki67, cells were fixed and permeabilized using a Fix/Permeabilization kit (eBioscience) and stained with anti-Ki67 mAb (REA183, Miltenyi Biotec).

### 4.5. Cell Isolation from Spleen

Spleens were harvested at indicated time points and analyzed for infiltration of human immune cells. Organs were homogenized through a cell strainer (100 µm; BD Biosciences). Erythrocytes were lysed and single-cell preparations were used for flow cytometry or restimulation with Phorbol 12-myristate 13-acetate (PMA)/Ionomycin.

### 4.6. Isolation of T Cell Subsets

CD4^+^CD25^+^Foxp3^+^ Treg were isolated from PBMC using anti-CD25 MicroBeads (Miltenyi Biotec) and depleted of contaminating CD8^+^, CD14^+^ and CD19^+^ cells with Dynabeads (Invitrogen, Hamburg, Germany) as described previously [49]. Purity was routinely >80%, Treg functionality was ensured in standard suppressor assays. For some experiments, PBMC were depleted of CD25 using corresponding Dynabeads (1 bead/cell; Invitrogen).

### 4.7. Cytokine Analysis

CD25-depleted PBMC from HC or MS patients were cultured in presence or absence of Treg from independent healthy donors (ratio 1:1) and stimulated with 0.5 µg/mL anti-CD3 mAb (clone OKT3, Bio X Cell, West Lebanon, NH, USA). Supernatants were collected 72 h after stimulation. Cytokines were measured by Cytometric Bead Array (BD Biosciences) following manufacturer’s instructions and analyzed by GraphPad Prism6 (Statcon, Witzenhausen, Germany). For intracellular cytokine staining, anti-IFN-γ mAb (BD Biosciences) was used. Spleen cells from humanized mice were restimulated with 1 µg/mL Ionomycin and 1 ng/mL PMA for 5 h, 4 h in the presence of Monensin (1.3 µM/mL). Afterwards, cells were fixed and permeabilized (perm/fix solution; BD Pharmingen) and stained for IFN-γ.

### 4.8. Suppressor Assays

Treg-depleted PBMC (10^5^ cells) were stimulated with 0.5 µg/mL anti-CD3 mAb (clone OKT3) and cultured in presence or absence of different Treg ratios (Treg:Teff 1:1 to 1:8) [44,49]. At day three, Teff proliferation was measured by ^3^H-Tdr (37 kBq/well) incorporation for an additional 16 h using a liquid β-scintillation counter.

### 4.9. Statistical Analysis

Results represent means ± SEM or SD. Statistical significance was determined using unpaired Student’s *t* test relative to control group (as indicated). *p*-values of less than 0.05 were considered significant and indicated in the corresponding figures (* *p* < 0.05; ** *p* < 0.01, **** *p* < 0.0001).

## 5. Conclusions

In conclusion, we demonstrated that DMF therapy modulates Treg resistance in RRMS patients. Similar to IFN-β, DMF improved responsiveness towards Treg-mediated suppression and significantly reduced IL-6R expression on peripheral T cells. These immune-modifying effects of DMF could be confirmed in a preclinical humanized mouse model in vivo.

## Figures and Tables

**Table 1:** Clinical characteristics of multiple sclerosis (MS) patients in the present study.

Sex	Age (year)	Disease Course	Disease Duration (year)	Treatment	Level of Disability (EDSS)	State
M	28.90	RRMS	7.87	Tecfidera	0.00	Remission
M	30.16	RRMS	0.72	Tecfidera	1.00	Relapse
W	45.74	RRMS	22.89	Tecfidera	5.00	Remission
W	45.89	RRMS	2.78	Tecfidera	1.00	Remission
W	33.30	RRMS	3.67	Tecfidera	4.50	Remission
W	43.11	RRMS	21.94	Tecfidera	2.50	Remission
M	32.55	RRMS	5.69	Tecfidera	0.00	Remission
W	42.45	RRMS	3.05	Tecfidera	0.00	Remission
M	23.28	RRMS	0.80	Tecfidera	2.00	Remission
W	50.99	RRMS	14.00	Tecfidera	1.00	Remission
W	30.93	RRMS	4.35	Tecfidera	4.00	Remission
M	19.13	RRMS	1.62	Tecfidera	0.00	Remission
W	54.45	RRMS	2.54	Tecfidera	1.00	Remission
M	25.98	RRMS	2.40	Tecfidera	0.00	Remission
M	31.62	RRMS	8.26	Tecfidera	1.00	Relapse
W	51.70	RRMS	20.09	Tecfidera	2.00	Remission
W	26.15	RRMS	11.10	Tecfidera	2.00	Remission
W	22.25	RRMS	3.70	Tecfidera	0.00	Remission
W	29.85	RRMS	1.55	Tecfidera	0.00	Remission
W	35.88	RRMS	3.26	Tecfidera	1.00	Remission
M	26.15	RRMS	3.07	Tecfidera	3.00	Remission
W	20.05	RRMS	1.16	Tecfidera	0.00	Remission
M	37.88	RRMS	6.17	Tecfidera	1.00	Remission
W	36.32	RRMS	11.43	Tecfidera	3.00	Remission
M	49.81	RRMS	18.44	Tecfidera	6.00	Remission
W	63.94	RRMS	0.15	untreated	2.00	Relapse
M	57.96	RRMS	0.42	untreated	1.00	Remission
W	56.00	RRMS	6.80	untreated	2.00	Remission
W	51.96	RRMS	1.33	untreated	1.50	Remission
M	23.20	RRMS	0.72	untreated	2.00	Remission
W	41.11	RRMS	0.57	untreated	3.00	Remission
W	27.56	RRMS	4.59	untreated	3.00	Relapse
W	63.75	PPMS	3.03	untreated	3.00	Remission
W	18.81	RRMS	9.22	untreated	1.00	Relapse
M	41.22	RRMS	8.98	untreated	2.00	Relapse
W	41.66	CIS	1.19	untreated	2.00	Remission
W	21.54	RRMS	5.04	untreated	1.00	Relapse
W	24.22	RRMS	0.29	untreated	1.00	Remission
W	48.59	CIS	1.14	untreated	2.00	Remission
W	41.74	CIS	1.27	untreated	2.00	Remission
W	37.41	RRMS	6.10	untreated	1.00	Remission
M	45.38	RRMS	24.10	untreated	3.00	Remission
W	40.66	RRMS	0.53	untreated	1.00	Remission
W	51.08	RRMS	5.29	untreated	3.50	Remission
W	37.54	RRMS	13.18	untreated	1.00	Relapse
M	34.71	RRMS	18.21	untreated	1.00	Remission
W	56.07	RRMS	15.23	untreated	3.00	Relapse
M	45.20	RRMS	15.25	untreated	1.00	Remission
W	51.85	RRMS	2.47	untreated	1.00	Remission
M	46.15	RRMS	5.44	untreated	1.00	Relapse
W	48.54	RRMS	5.45	untreated	1.00	Remission
W	45.04	RRMS	12.52	untreated	2.00	Remission

Peripheral blood mononuclear cells (PBMC) were collected in heparinized tubes from 49 relapse-remitting MS (RRMS) patients (age 18 to 63 years) and three with a clinically isolated syndrome (CIS, age 41 to 48 years). Ten RRMS patients showed a relapse, the remaining patients were in remission. The Expanded Disability Status Scale (EDSS) was used to quantify disability (0–6). Twenty-five patients were treated for at least four months with dimethyl fumarate (DMF, Tecfidera). Others had not received previous treatment six months before time point of analysis and were clinically stable.

**Table 2:** Total number of spleen cells and amounts of human CD45^+^ immune cells in PBMC-engrafted mice.

	HC	HC + Treg	MS	MS + Treg	DMF	DMF + Treg
spleen cells ×10^6^	52.41 ± 14.34	32.09 ± 4.85	41.67 ± 6.47	34.70 ± 9.95	57.38 ± 10.09	59.75 ± 9.65
huCD45^+^ cells in %	5.77 ± 3.89	1.77 ± 0.28	3.87 ± 0.52	2.80 ± 0.86	7.41 ± 3.06	0.79 ± 0.15

Mice transferred with human PBMC ± Treg were sacrificed on day eight. Number of spleen cells was determined and ratios of human CD45^+^ immune cells were analyzed by flow cytometry. Data are displayed as mean values ± SEM of *n* = 5 mice per group.
